# Poisoning of Cattle through a Licking-stone (Salt-poisoning)

**Published:** 1897-01

**Authors:** 


POISONING OF CATTLE THROUGH A LICKING-STONE (SALT-POISONING). Horn, in the *Wochenschrift für Tierheilkunde*, states that a farmer turned his herd of cattle, consisting of 9 cows, 14 calves, and 2 oxen, out to graze one morning in the late autumn, and left them there the whole day. The troughs in the stalls were then thoroughly cleansed and filled with fresh water. A licking-stone which had been placed by the milkmaid on the edge of the troughs where the cows drank fell into the water through some accident, and during the day entirely dissolved. In the evening the cows eagerly drank the water. Toward 9 o'clock the owner heard a noise in the stable, and when he went in found one cow lying motionless on the floor. While he was killing this one a second and third cow fell, and six others acted as if they were intoxicated. An investigation made by a veterinarian during the night had the following result: Partial and full loss of consciousness on the part of the sick cows, signs of intoxication, reddening of the conjunctiva, paunch slightly swollen, contraction toward the rectum, which could easily be pressed out in the animals more severely attacked; a hardly perceptible, weak and slow pulse; both urination and evacuation hindered; no fever. Three other animals, slain during the night showed no pathological change except a slight reddening of the mucous membrane of the stomach. Four cows which had not been so severely attacked recovered slowly after six hours, but showed twice a giddy, sleepy condition. In one cow the state of intoxication lasted the next day and night, the animal finally recovering. Another cow suddenly showed complete lameness of the shoulder-muscles on the left side.—*Schweizer Archiv.* January and February, 1896.

